# Beyond bone density: toward an integrated bone– muscle–function framework for fragility fracture prevention

**DOI:** 10.3389/fmed.2026.1881899

**Published:** 2026-08-05

**Authors:** Shengzhu Lu, Chaodong Liu, Yinyao Gou, Hao Liu

**Affiliations:** 1 Department of Orthopaedics, Wuchang Hospital Affiliated to Wuhan University of Science and Technology, Wuhan, Hubei, China; 2 Faculty of Engineering, The University of Sydney, Camperdown, NSW, Australia

**Keywords:** fragility fracture, frailty, osteoporosis, prevention, sarcopenia

## Abstract

Fragility fractures are a major clinical and public health challenge in aging societies. Current prevention strategies are commonly organized around skeletal risk assessment and osteoporosis management, yet fracture occurrence and post-fracture outcomes in older adults are also influenced by muscle dysfunction, frailty, fall propensity, physiological reserve, and rehabilitation capacity. This Perspective proposes a bone–muscle–function framework to complement existing osteoporosis-centered approaches to fragility fracture prevention. In this framework, bone represents skeletal strength and structural fragility, muscle represents sarcopenia-related impairment in strength and physical performance, and function represents frailty-related reserve, resilience, fall propensity, and recovery capacity. Osteosarcopenia provides the bone–muscle foundation, whereas the addition of function extends the framework to include broader vulnerability and recovery potential. Rather than simply adding frailty and sarcopenia to osteoporosis assessment, it organizes bone, muscle, and function into a staged framework for risk identification, recovery planning, and secondary prevention.

## 1 Introduction

Fragility fractures represent a major clinical and public health challenge in aging societies. Hip, vertebral, distal radius, and other low-trauma fractures are associated with substantial pain, disability, loss of independence, increased care needs, recurrent fractures, and excess mortality, particularly among older adults (1–3). As population aging accelerates worldwide, fragility fracture prevention is no longer a concern of osteoporosis care or orthopedics alone, but an increasingly important multidisciplinary priority spanning geriatrics, rehabilitation medicine, endocrinology, nutrition, nursing, public health, sports medicine, and sport science, particularly in relation to primary prevention through strength training, physical conditioning, and fall-risk reduction (4, 5).

Current osteoporosis and fragility fracture guidelines provide an evidence-based foundation for prevention through clinical risk factor assessment, bone mineral density (BMD) measurement, FRAX-based fracture risk estimation, vertebral fracture assessment when indicated, and pharmacological and non-pharmacological interventions (6). These approaches remain essential for identifying and managing individuals at high risk of fragility fractures. Although contemporary guidelines increasingly acknowledge falls, exercise, nutrition, rehabilitation, and multidisciplinary care, routine prevention pathways remain primarily organized around skeletal risk assessment and osteoporosis management. However, in older adults, fragility fracture vulnerability is not determined by bone strength alone. Many older adults who sustain fragility fractures present with multiple geriatric vulnerabilities, including frailty, sarcopenia, impaired balance, reduced gait speed, malnutrition, cognitive impairment, and limited rehabilitation capacity (7–10). These non-skeletal factors may increase fall propensity, impair protective responses during a fall, delay post-fracture functional recovery, and contribute to recurrent fragility fractures.

This gap highlights the limitations of a predominantly skeleton-centered prevention paradigm. BMD provides essential information about skeletal strength, but it does not fully capture whole-body vulnerability in older adults. Frailty reflects reduced physiological reserve and diminished resilience to stressors, whereas sarcopenia is characterized by impairments in muscle strength, mobility, balance, and physical performance (11, 12). Together, frailty and sarcopenia may serve as clinically actionable markers linking osteoporosis, falls, post-fracture functional decline, and recurrent fragility fracture risk. The emerging concept of osteosarcopenia further emphasizes that osteoporosis and sarcopenia frequently coexist and may share biological pathways, functional consequences, and clinical risk factors rather than representing isolated aging-related conditions (13, 14).

This Perspective aims to outline how frailty and sarcopenia can be integrated into fragility fracture prevention. We first describe fragility fracture vulnerability as a multidimensional risk state, then examine frailty and sarcopenia as two clinically relevant dimensions of whole-body vulnerability. We further introduce osteosarcopenia as a conceptual bridge linking bone loss and muscle decline. Building on this concept, our ultimate goal is to propose a bone–muscle–function framework that extends existing osteoporosis-centered prevention by connecting skeletal risk assessment with muscle function, frailty screening, fall-risk stratification, rehabilitation planning, and secondary fracture prevention. This framework seeks to move fragility fracture prevention from a predominantly skeleton-centered framework toward a multidisciplinary, patient-centered approach that better addresses the complex vulnerability of older adults.

## 2 Multidimensional vulnerability in fragility fracture prevention

### 2.1 Fragility fracture vulnerability as a multidimensional risk state

Although reduced bone strength is central to the pathogenesis of fragility fractures, the clinical pathway leading to fracture is not uniform across older adults (15, 16). Reduced BMD reflects loss of bone mass, while deterioration in bone microarchitecture, cortical porosity, trabecular architecture, and material properties may further compromise skeletal strength, explaining why osteoporosis remains a central target for fracture prevention (17). However, skeletal fragility alone does not fully determine whether a low-trauma event results in fracture. Some individuals sustain fractures mainly in the context of marked skeletal weakness, whereas others experience fractures when moderate skeletal compromise is compounded by recurrent falls, impaired balance, reduced mobility, poor nutrition, cognitive impairment, or limited functional reserve (18–20). In addition, fall direction, impact characteristics, neuromuscular control, and protective responses during a fall may influence whether skeletal vulnerability is translated into an actual fracture (21). After fracture occurrence, recovery is further shaped by functional reserve, cognition, nutritional status, and rehabilitation potential (22, 23).

These layered influences create substantial heterogeneity among older adults who may appear similar when assessed only by skeletal measures. Some may remain relatively robust despite low BMD, whereas others may have only moderate skeletal risk but substantial whole-body vulnerability (24–26). These vulnerabilities often coexist and may shape not only fracture occurrence but also post-fracture functional decline, dependence, and recurrent fragility fractures. Consequently, fragility fracture vulnerability should be understood as a multidimensional risk state rather than a skeletal condition alone.

### 2.2 Frailty as a marker of whole-body vulnerability

Frailty provides a clinical lens for understanding the patient-level vulnerability that shapes fracture risk and recovery in older adults. Unlike BMD, which primarily reflects bone strength, frailty captures multidimensional reductions in physiological reserve, including impairments in mobility, nutrition, cognition, endurance, balance, and resilience to acute stressors (11, 27, 28). In the context of fragility fractures, this whole-body vulnerability is highly relevant because fractures often result from the interaction between fragile bone and impaired physical function rather than from skeletal weakness alone (24).

Accumulating evidence suggests that frailty is closely associated with both falls and fractures in older adults. A systematic review and meta-analysis identified frailty as a risk factor for falls, supporting its relevance to the upstream pathway of fall-related injury and fracture risk (29). Prospective studies have further linked frailty status with a higher risk of incident fractures in older adults (26, 30). After fracture occurrence, frailty has also been associated with poorer recovery trajectories, including postoperative complications, functional decline, fracture-related readmission, and mortality. In one cohort of patients aged ≥75 years undergoing hip fracture surgery, frail patients had higher 1-year mortality than non-frail patients (21.3 vs. 4.4%), and frailty remained independently associated with mortality (adjusted HR, 3.88; 95% CI, 1.28–11.77) (31). In a linked-data cohort of 28,567 older hip fracture patients, intermediate and high frailty risk were associated with higher risks of fracture-related readmission and death than low frailty risk (32). Collectively, these data position frailty as more than a background geriatric condition; it is a marker of both fracture susceptibility and post-fracture prognosis.

**Figure 1:**
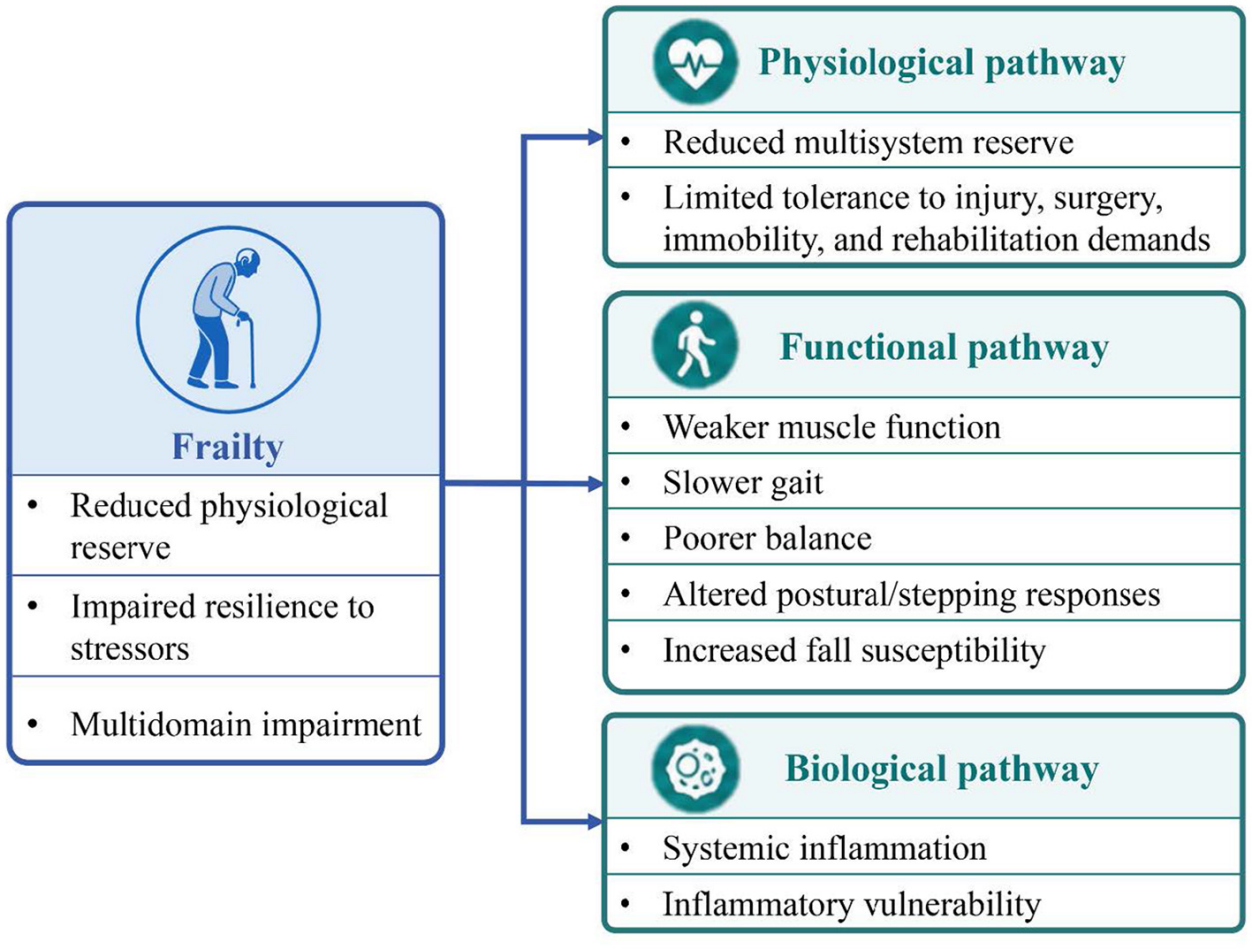
Mechanistic pathways linking frailty to fragility fracture vulnerability.

The clinical relevance of frailty may be explained by several converging pathways. At the physiological level, frailty reflects reduced multisystem reserve and impaired resilience to stressors, which may limit recovery when older adults face injury, surgery, immobility, or rehabilitation demands (33–35). At the functional level, frailty is associated with measurable sensory and motor deficits, including weaker muscle function, slower gait, poorer balance, and altered postural or stepping responses (36, 37). At the biological level, systemic inflammation may partly mediate the association between frailty and incident fractures, indicating that frailty may reflect not only functional impairment but also underlying inflammatory vulnerability (30, 38). These pathways help explain how frailty contributes to fracture susceptibility and post-fracture functional decline within a single clinical trajectory. The mechanisms are summarized in Figure 1.

For prevention, the value of frailty lies in its ability to translate diffuse vulnerability into measurable risk domains. Frailty scales and comprehensive geriatric assessment can help identify older adults with reduced physiological reserve across medical, cognitive, nutritional, and functional domains, while simple performance measures such as gait speed, grip strength, chair-stand performance, and balance testing provide practical indicators of mobility, muscle strength, and physical performance (39, 40). These tools may identify older adults whose fracture risk is shaped by reduced reserve, impaired mobility, and limited recovery capacity. Therefore, frailty assessment can complement skeletal risk evaluation by informing individualized prevention, rehabilitation planning, and multidisciplinary follow-up.

### 2.3 Sarcopenia and muscle function as overlooked determinants of fracture risk

While frailty captures whole-body vulnerability, sarcopenia focuses attention on the muscular and performance-related components of fracture risk. Characterized by low muscle strength, reduced muscle quantity or quality, and impaired physical performance, sarcopenia affects several functions that are directly relevant to fracture prevention, including mobility, gait stability, balance control, fall avoidance, and the ability to respond effectively when a fall occurs (41, 42). In this sense, sarcopenia is not only a disorder of muscle mass or strength, but also a functional condition that may influence whether skeletal fragility is translated into an actual fracture (42–44).

Evidence increasingly supports the association between sarcopenia, impaired muscle function, and fracture risk in older adults. In a UK Biobank study of 387,025 participants, sarcopenia was associated with higher incident fracture risk (adjusted HR, 1.30; 95% CI, 1.08–1.56) (45). In older Swedish women, sarcopenia defined by muscle function/strength criteria was associated with increased risks of any fracture (HR, 1.48; 95% CI, 1.10–1.99) and major osteoporotic fracture (HR, 1.42; 95% CI, 1.03–1.98) (46). A CHARLS-based longitudinal study further showed that older Chinese men with both handgrip strength weakness and asymmetry had increased odds of incident hip fracture (adjusted OR, 2.31; 95% CI, 1.17–4.52) (44). Together, these studies suggest that muscle impairment contributes to fracture vulnerability through pathways that extend beyond bone mass alone.

**Figure 2:**
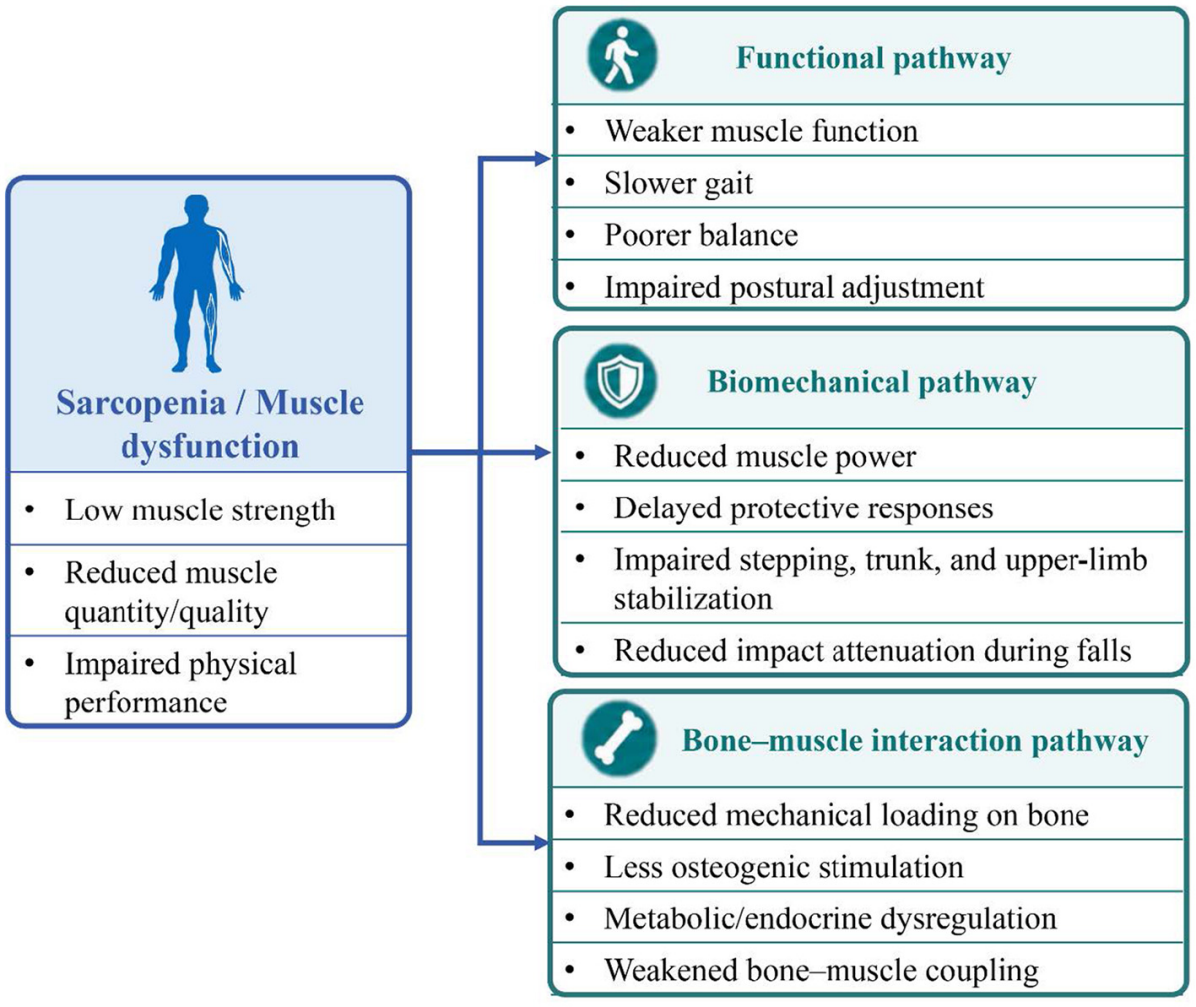
Mechanistic pathways linking sarcopenia to fragility fracture vulnerability.

The pathways linking sarcopenia to fracture vulnerability are more directly related to biomechanics, metabolism, and physical performance than those linking frailty to fracture risk. Reduced muscle strength and impaired physical performance can compromise gait stability, balance control, and postural adjustment, thereby increasing fall susceptibility (41, 42, 47, 48). During a fall, reduced muscle power and delayed protective responses may limit the ability to stabilize the body, generate stepping reactions, coordinate upper-limb or trunk responses, or attenuate impact forces, increasing the likelihood that skeletal vulnerability is translated into fracture (21, 49, 50). In parallel, skeletal muscle contributes to bone health through mechanical loading; therefore, loss of muscle mass or strength may reduce osteogenic mechanical stimulation and weaken the functional coupling between muscle and skeletal adaptation (51–54). In addition to mechanical loading, sarcopenia may also compromise bone health through metabolic and endocrine pathways. Skeletal muscle is increasingly recognized as a metabolic and endocrine organ that communicates with bone through myokines and other humoral mediators (55, 56). Loss of muscle mass may reduce beneficial muscle-derived signaling to bone, while sarcopenia-related insulin resistance, chronic low-grade inflammation, mitochondrial dysfunction, and impaired protein and energy metabolism may further disturb bone remodeling and weaken muscle–bone crosstalk (57, 58). After fracture occurrence, sarcopenia may further impair recovery by limiting early mobility, reducing rehabilitation responsiveness, and delaying recovery of independent ambulation (59–61). These mechanisms are summarized in Figure 2.

The clinical importance of sarcopenia lies in its measurability and modifiability. Simple measures such as handgrip strength, chair-stand performance, gait speed, balance testing, and muscle mass assessment are commonly used to evaluate the core domains of sarcopenia, including muscle strength, muscle quantity or quality, and physical performance (12, 62). Incorporating these measures into fracture risk evaluation may help identify older adults whose vulnerability is driven not only by bone loss but also by impaired muscle function and physical performance. Identifying sarcopenia may also guide resistance exercise, balance training, nutritional optimization, and structured rehabilitation, thereby connecting fracture risk evaluation with interventions that target falls, mobility, and post-fracture recovery.

### 2.4 Osteosarcopenia as a bridge between bone and muscle decline

The concept of osteosarcopenia brings the skeletal and muscular dimensions of fracture vulnerability into a single framework. It refers to the coexistence of osteoporosis or osteopenia and sarcopenia in the same individual (63–65). This concept is particularly relevant to fragility fracture prevention because it emphasizes that low bone strength and impaired muscle function may jointly contribute to fracture vulnerability rather than acting as isolated age-related conditions.

The value of osteosarcopenia lies in its recognition of bone–muscle interaction. Skeletal muscle contributes to bone health through mechanical loading and mechanotransduction, while muscle strength, gait stability, and balance influence fall risk, fall biomechanics, and impact protection (21, 52, 54). When skeletal fragility coexists with impaired muscle function, fall-related impact forces are more likely to result in fracture because weakened bone is less able to tolerate mechanical loading. In parallel, shared aging-related mechanisms, including physical inactivity, malnutrition, chronic low-grade inflammation, hormonal changes, and metabolic dysregulation, may contribute to the concurrent decline of bone and muscle (14). Therefore, osteosarcopenia provides a useful bridge between osteoporosis-centered and sarcopenia-centered accounts of fracture vulnerability.

However, osteosarcopenia alone does not fully capture the broader functional vulnerability that shapes fragility fracture risk and recovery. Even when bone loss and muscle impairment coexist, differences in physiological reserve, resilience, and recovery capacity may still lead to markedly different clinical trajectories (24–26). This is where frailty becomes essential: it extends the bone–muscle perspective by representing whole-body vulnerability and helping to explain why similar skeletal and muscular deficits may result in different fall, fracture, and recovery outcomes.

Taken together, frailty, sarcopenia, and osteosarcopenia should not be viewed as competing concepts. Sarcopenia highlights muscle dysfunction and physical performance; osteosarcopenia links bone loss with muscle decline; and frailty adds the broader dimension of reserve, resilience, and recovery capacity. However, these concepts remain fragmented when considered separately. This provides the rationale for a bone–muscle–function framework that organizes skeletal fragility, muscle impairment, and functional reserve into an integrated structure for fragility fracture prevention.

## 3 From osteosarcopenia to a bone–muscle–function framework

**Figure 3:**
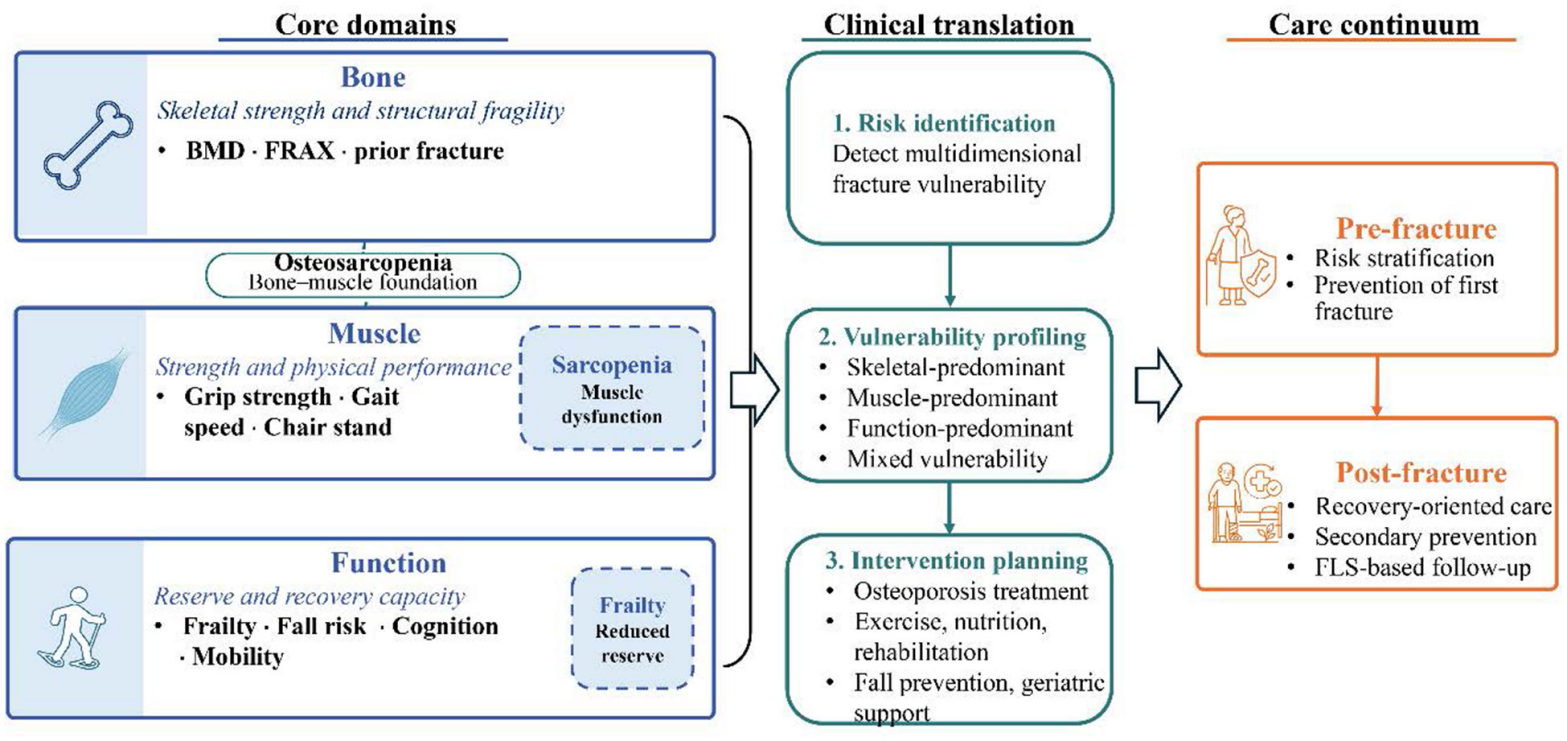
Conceptual and clinical translation of the bone–muscle–function framework for fragility fracture prevention. The framework organizes bone, muscle, and function as interrelated domains that inform risk identification, vulnerability profiling, and intervention planning across pre-fracture and post-fracture care.

Building on the preceding discussion, this Perspective proposes extending the osteosarcopenia concept into a bone–muscle–function framework of fragility fracture prevention (Figure 3). In this framework, bone refers to tissue-level skeletal fragility, including reduced bone strength and structural compromise; muscle refers to neuromuscular capacity, including muscle strength, muscle quantity or quality, physical performance, gait stability, balance, and impact-protection capacity; and function refers to the integrated patient-level expression of reserve, mobility, fall vulnerability, rehabilitation participation, and recovery potential in real-world settings. Function therefore does not simply duplicate sarcopenia or frailty. Rather, it represents the broader clinical domain through which skeletal and muscular impairments are translated into fracture occurrence, functional decline, and secondary prevention needs.

The conceptual contribution of this framework is not merely that it combines three familiar domains. Rather, it organizes bone, muscle, and function into a staged clinical structure that links pre-fracture vulnerability identification, fracture occurrence, post-fracture recovery, and secondary prevention within a single continuum. In doing so, the framework connects organ-level skeletal risk with patient-level vulnerability and provides a common structure for both prevention planning before fracture and care coordination after fracture. It is intended to complement, rather than replace, existing osteoporosis-centered approaches: skeletal risk assessment remains essential, but is situated within a broader framework in which muscle impairment and functional reserve shape both fracture susceptibility and clinical outcomes.

This framework may be especially useful in older adults whose fracture vulnerability is not adequately explained by bone density alone. Some individuals may present with predominantly skeletal fragility, whereas others may have moderate skeletal compromise but marked muscle impairment, frailty, recurrent falls, or limited recovery capacity. A bone–muscle–function perspective may therefore help explain why patients with apparently similar skeletal risk can experience different fracture trajectories and post-fracture outcomes. By organizing these dimensions more explicitly, the framework provides a conceptual bridge between osteoporosis management, sarcopenia assessment, frailty-informed care, fall prevention, rehabilitation planning, and fracture liaison services.

## 4 Clinical translation of the bone–muscle–function framework

### 4.1 A staged prevention strategy based on bone, muscle, and function

The clinical value of the bone–muscle–function framework becomes most evident when it is translated into staged assessment and intervention across the fragility fracture care continuum. In this framework, bone captures tissue-level skeletal fragility, muscle captures neuromuscular capacity and physical performance, and function captures the integrated patient-level expression of reserve, fall vulnerability, rehabilitation capacity, and recovery potential. These domains are interrelated rather than mutually exclusive, but separating them conceptually may help clinicians organize assessment and intervention priorities more clearly.

Before fracture occurrence, the framework can support multidimensional risk identification in older adults who may not be adequately characterized by skeletal assessment alone. A practical first-step screen may include prior fragility fracture, BMD and/or FRAX-based skeletal risk estimation, simple measures of muscle strength or physical performance such as handgrip strength, chair-stand performance, or gait speed, and brief functional indicators such as fall history, mobility limitation, or frailty screening. Such an approach may help identify whether vulnerability is predominantly skeletal, muscle-related, function-related, or distributed across multiple domains.

Importantly, this staged structure may also help align vulnerability profiles with intervention priorities. Individuals with predominantly skeletal fragility may benefit most from timely osteoporosis treatment and fracture-risk monitoring, whereas those with substantial muscle impairment may require greater emphasis on resistance exercise, nutritional support, balance training, and rehabilitation. In contrast, those with marked functional vulnerability may require a broader geriatric approach incorporating fall prevention, medication review, mobility support, cognitive assessment, and coordinated multidisciplinary follow-up. In many older adults, these domains overlap, and the framework may therefore support more individualized combinations of intervention rather than a single uniform prevention pathway.

A practical summary of the framework across the fragility fracture care continuum, including representative clinical features, candidate assessment tools, and corresponding clinical implications across the bone, muscle, and function domains, is shown in Table 1.

**Table 1:** Clinical interpretation of the bone–muscle–function framework across the fragility fracture care continuum.

Domain	Core construct	Representative clinical features	Candidate assessment tools/measures	Main clinical implications
Bone	Skeletal strength and structural fragility	Low BMD, prior fragility fracture, vertebral fracture, deteriorated bone quality, high estimated fracture risk	DXA-derived BMD, FRAX, vertebral fracture assessment, fracture history, selected bone turnover markers or imaging when appropriate	Identification of skeletal fragility and guidance for initiation or optimization of anti-osteoporosis treatment and fracture-risk monitoring
Muscle	Muscle strength, muscle quantity/quality, and physical performance	Weak grip strength, slow chair rise, reduced gait speed, impaired balance, low muscle mass, sarcopenia	Handgrip strength, chair-stand test, gait speed, short physical performance battery, balance testing, appendicular lean mass or other muscle mass measures when available	Identification of sarcopenia-related vulnerability and guidance for resistance exercise, nutritional support, and targeted rehabilitation
Function	Integrated patient-level reserve, fall vulnerability, and recovery capacity	Frailty, recurrent falls, impaired mobility, limited endurance, cognitive impairment, polypharmacy, reduced resilience, poor rehabilitation tolerance	Frailty screening tools, fall history, ADL/IADL assessment, mobility assessment, medication review, cognition screening, comprehensive geriatric assessment when indicated	Identification of whole-body vulnerability and guidance for fall prevention, medication optimization, rehabilitation and discharge planning, multidisciplinary follow-up, and secondary fracture prevention

The three domains are interrelated rather than mutually exclusive. Bone captures tissue-level skeletal fragility, muscle captures neuromuscular capacity and physical performance, and function captures the integrated patient-level expression of reserve, fall vulnerability, and recovery potential. Together, these domains provide a staged structure for risk identification, rehabilitation planning, and secondary fracture prevention. BMD, bone mineral density; DXA, dual-energy X-ray absorptiometry; FRAX, Fracture Risk Assessment Tool; ADL, activities of daily living; IADL, instrumental activities of daily living.

### 4.2 Application of the framework after fracture: toward integrated secondary prevention

Once an index fragility fracture occurs, the prevention focus shifts from primary risk identification to integrated secondary prevention and recovery-oriented care. This transition provides a particularly relevant setting for application of the bone–muscle–function framework.

In the post-fracture setting, bone remains essential for confirming osteoporosis, estimating future fracture risk, and guiding anti-osteoporosis therapy. However, muscle and function become equally important for understanding early mobility, tolerance of rehabilitation, risk of recurrent falls, likelihood of functional recovery, and the degree of support required after discharge. A tri-domain assessment may therefore help clinicians move beyond the question of why the fracture occurred to the equally important questions of how recovery is likely to proceed and what type of secondary prevention strategy is needed.

Within this framework, the muscle domain may identify patients whose recovery is likely to be limited by sarcopenia-related weakness, poor physical performance, or impaired balance, thereby supporting early rehabilitation planning, mobility training, nutritional intervention, and progressive strengthening strategies. The function domain may identify patients with broader whole-body vulnerability, including frailty, recurrent falls, cognitive impairment, polypharmacy, or limited physiological reserve, all of which may affect postoperative recovery, discharge readiness, long-term independence, and recurrent fracture risk. In these patients, comprehensive geriatric assessment and multidisciplinary coordination may be particularly important, as supported by evidence from comprehensive geriatric and orthogeriatric co-management studies (66–69).

Fracture liaison services (FLS) provide a practical clinical setting in which this framework may be implemented. FLS are coordinated, multidisciplinary care models that systematically identify patients with fragility fractures and link them to osteoporosis assessment, treatment, and secondary fracture prevention (70, 71). By creating a structured pathway after an index fracture, FLS have become an important strategy for reducing missed opportunities in osteoporosis assessment, treatment initiation, adherence, and secondary fracture prevention (72–75). Traditionally, the FLS model has focused on identifying osteoporosis after fracture and improving initiation of anti-fracture treatment. The bone–muscle–function framework suggests that FLS could also serve as a structured platform for broader secondary prevention by incorporating selected measures of muscle strength, physical performance, frailty, fall history, cognition, and functional reserve into routine post-fracture evaluation. Such an approach may improve patient stratification, guide referral to rehabilitation or geriatric services, and support more individualized secondary prevention pathways.

In this sense, the framework extends FLS from a predominantly skeletal post-fracture pathway toward a more integrated strategy that addresses recurrent fractures, functional decline, and recovery potential together. This broader application is not intended to diminish the central role of osteoporosis care, but to ensure that bone-targeted treatment is coordinated with fall-risk reduction, muscle and functional recovery, rehabilitation planning, and long-term maintenance of independence.

## 5 Conclusion

This Perspective proposes a bone–muscle–function framework as a complementary approach to fragility fracture prevention. BMD assessment, fracture risk estimation, vertebral fracture evaluation, and anti-osteoporosis therapy remain essential components of osteoporosis care. However, older adults' fracture vulnerability is also shaped by muscle dysfunction, frailty, fall propensity, physiological reserve, and recovery capacity. Osteosarcopenia provides the bone–muscle foundation of this framework, whereas the addition of function incorporates frailty-related reserve, resilience, and rehabilitation potential.

Future research should validate this framework in prospective cohorts and intervention studies, clarify how these domains interact to predict fracture and recovery outcomes, and test whether staged, vulnerability-based prevention strategies can improve both skeletal and patient-centered outcomes. In clinical practice, settings such as FLS may provide a feasible platform for implementing and evaluating integrated bone–muscle–function pathways after an index fracture. Ultimately, fragility fracture prevention should move from identifying fragile bones alone toward identifying fragile patients, supporting more individualized and patient-centered care for older adults.

## Data Availability

The original contributions presented in the study are included in the article/supplementary material, further inquiries can be directed to the corresponding author.
